# Integrated analysis of mRNA and miRNA expression in response to interleukin-6 in hepatocytes

**DOI:** 10.1016/j.dib.2015.05.023

**Published:** 2015-06-10

**Authors:** Samuel W. Lukowski, Richard J. Fish, Juliette Martin-Levilain, Carmen Gonelle-Gispert, Leo H. Bühler, Pierre Maechler, Emmanouil T. Dermitzakis, Marguerite Neerman-Arbez

**Affiliations:** a Department of Genetic Medicine and Development, University of Geneva Medical School, 1211 Geneva, Switzerland; b Institute of Genetics and Genomics in Geneva (iGE3), 1211 Geneva, Switzerland; c Department of Cell Physiology and Metabolism, University of Geneva Medical Center, Geneva, Switzerland; d Surgical Research Unit, Department of Surgery, University Hospital, 1211 Geneva, Switzerland; e Swiss Institute of Bioinformatics, 1211 Geneva, Switzerland

## Abstract

Understanding the interactions between miRNAs and genes they regulate during the acute phase response is crucial to our understanding of inflammatory diseases and processes. Inducing the acute phase response in hepatocytes by stimulating them with interleukin-6 [1] and then examining global changes in mRNA and miRNA expression can provide insight into the timing and dynamics of these interactions. Here we provide additional data for our study, Ref. [2]. In this data, we identify and validate IL-6-induced changes in gene expression [3–6] and their functional relationships over time and between cell types by gene ontology [7,8]. We also provide data showing the enrichment of miRNA binding motifs in the 3׳UTRs of differentially expressed genes [9], and their predicted gene targets derived from our RNA-seq data [10].

## 1 Specifications table

**Table:** 

Subject area	Biology
More specific subject area	HepG2, human and mouse hepatocyte mRNA and miRNA transcriptome
Type of data	Tables, graphs, image
How data was acquired	RNA-seq using Illumina Hi-Seq 2000, qPCR, western blot
Data format	Analyzed
Experimental factors	Hepatocytes were either untreated or stimulated with interleukin-6, and mRNA and small RNA-seq libraries were generated for untreated, 6 h and 24 h post-IL-6 timepoints.
Experimental features	Samples were HepG2 cells, human primary hepatocytes derived from healthy liver tissue, and mouse hepatocytes were derived from healthy mice with a mixed 129 Sv/C57Bl6J genetic background.
Data source location	Geneva, Switzerland
Data accessibility	Data is available with the article

### 1.1 Value of the data

• This data provides an integrated analysis of miRNA and mRNA expression in a model of the acute phase response in human and mouse hepatocytes.
• We investigated mRNA and miRNA expression between cell types after IL-6 stimulation.
• We observed a delayed response in gene expression changes in mouse hepatocytes compared to HepG2 cells and human hepatocytes. This is also reflected in the gene ontology and pathways analyses.
• We identified a subset of differentially expressed miRNAs that regulate the expression of important acute phase response genes at specific time points, and in different hepatocyte models, following induction of the IL-6-mediated acute phase response.

## 2 Data, experimental design, materials and methods

Figure 1: Top 20 up- and down-regulated genes in HepG2 (A–E), human primary hepatocytes (F–J) and mouse primary hepatocytes (K–O) between 0–6 h, 0–24 h, 6–24 h, 6 h±IL-6 and 24 h±IL-6. Time zero=untreated cells. Plotted values are the log2 fold-change of gene expression.

Figure 2: ConsensusPathDB (CPdB) analyses of GO terms and enriched pathways in HepG2 cells. The *p*-value cutoff was 0.01 and the minimum input overlap was 2.

Figure 3: CPdB analyses of GO terms and enriched pathways in human primary hepatocytes. The *p*-value cutoff was 0.01 and the minimum input overlap was 2.

Figure 4: CPdB analyses of GO terms and enriched pathways in mouse primary hepatocytes. The *p*-value cutoff was 0.01 and the minimum input overlap was 2.

Figure 5: Heatmap of mean expression (log2 RPKM) of the 0–24 h intersection genes in all three cell types (23 genes), with expression data for untreated (UT), 6 h and 24 h post-IL-6 induction – see Figure 1 in Ref. [2].

Figure 6: Validation of RNA-seq data (RPKM) by qPCR and western blot. Fibrinogen mRNA expression (*FGA*, *FGB*, *FGG*) was validated using qPCR in human primary hepatocytes (A and B) and mouse primary hepatocytes (C and D). Gene expression is expressed as a percentage of the IL-6 untreated control (black bars). The time zero data in the IL-6-positive samples is also untreated. Panel E shows a western blot of secreted fibrinogen protein, reduced to the individual chains (Aα, Bβ, and Ɣ), in conditioned media from human primary hepatocyte cultures that were either untreated or treated with IL-6 for 6 h or 24 h. The protein control is purified fibrinogen preparation, and the loading control is secreted albumin in the conditioned media.

Table 1: Differentially expressed miRNAs in IL-6-stimulated hepatocytes (heatmap data, untransformed cpm values – see Figure 4 in Ref. [2]).

Table 2: Significant miRNAs binding to over-represented 8nt motif in up- or down-regulated DE mRNA targets. *P*-value is the significance of the complementarity between the Weeder-calculated sequence motif and the miRNA seed. Asterisk represents the star-arm of miRNA precursor.

Table 3: Hypergeometric analysis of DE miRNAs and their up- or down-regulated, differentially expressed TargetScan-predicted targets. Entries in bold text are statistically significant (*P*<0.05).

Table 4: Up-regulated DE mRNA targets of down-regulated DE miRNAs in HepG2 cells.

Table 5: Up-regulated DE mRNA targets of down-regulated DE miRNAs in human primary hepatocytes.

Table 6: Up-regulated DE mRNA targets of down-regulated DE miRNAs in mouse primary hepatocytes.

## References

[1] Baumann H., Prowse K.R., Marinković S., Won K.A., Jahreis G.P. (1989). Stimulation of hepatic acute phase response by cytokines and glucocorticoids. Ann. N. Y. Acad. Sci..

[2] Lukowski S.W., Fish R.J., Martin-Levilain J., Gonelle-Gispert C., Bühler L.H., Maechler P., Dermitzakis E.T., Neerman-Arbez M. (2015). Integrated analysis of mRNA and miRNA expression in response to interleukin-6 in hepatocytes. Genomics.

[3] Anders S., Pyl P.T., Huber W. (2015). HTSeq–a Python framework to work with high-throughput sequencing data. Bioinformatics.

[4] Harrow J., Frankish A., Gonzalez J.M., Tapanari E., Diekhans M., Kokocinski F., Aken B.L., Barrell D., Zadissa A., Searle S. (2012). GENCODE: the reference human genome annotation for The ENCODE Project. Genome Res..

[5] Kim D., Pertea G., Trapnell C., Pimentel H., Kelley R., Salzberg S.L. (2013). TopHat2: accurate alignment of transcriptomes in the presence of insertions, deletions and gene fusions. Genome Biol..

[6] Robinson M.D., McCarthy D.J., Smyth G.K. (2010). edgeR: a Bioconductor package for differential expression analysis of digital gene expression data. Bioinformatics.

[7] Kamburov A., Stelzl U., Lehrach H., Herwig R. (2013). The ConsensusPathDB interaction database: 2013 update. Nucleic Acids Res..

[8] Kamburov A., Wierling C., Lehrach H., Herwig R. (2009). ConsensusPathDB–a database for integrating human functional interaction networks. Nucleic Acids Res..

[9] Plaisier C.L., Bare J.C., Baliga N.S. (2011). miRvestigator: web application to identify miRNAs responsible for co-regulated gene expression patterns discovered through transcriptome profiling. Nucleic Acids Res..

[10] Lewis B.P., Burge C.B., Bartel D.P. (2005). Conserved seed pairing, often flanked by adenosines, indicates that thousands of human genes are microRNA targets. Cell.

