# Supplementary material for: Integrated analysis of mRNA and miRNA expression in response to interleukin-6 in hepatocytes
Source: Data Brief. 2015 Jun 10;4:226–8. doi: 10.1016/j.dib.2015.05.023 (PMC4510544; doi:10.1016/j.dib.2015.05.023)
Supplement: Supplementary file 1 — Supplementary data [file mmc1.zip › Supplementary Table 1.docx]

**Table 1:** Differentially expressed miRNAs in IL-6-stimulated hepatocytes (heatmap data, untransformed cpm values)

|  | **HepG2** | | | **Human** | | | **Mouse** | | |
| --- | --- | --- | --- | --- | --- | --- | --- | --- | --- |
|  | **UT** | **6h** | **24h** | **UT** | **6h** | **24h** | **UT** | **6h** | **24h** |
| **hsa-miR-126-5p** | 59.0 | 70.2 | 180.2 | 3051.6 | 4500.7 | 5580.4 | 30409.9 | 29687.7 | 14483.8 |
| **hsa-miR-1286** | 6.7 | 9.3 | 11.6 | 2.1 | 1.7 | 15.0 | n.d. | n.d. | n.d. |
| **hsa-miR-17-5p** | 18169.8 | 18616.5 | 22489.1 | 376.6 | 561.0 | 873.4 | 385.8 | 699.8 | 547.1 |
| **hsa-miR-181a-3p** | 132.9 | 122.2 | 155.8 | 88.6 | 120.4 | 122.8 | 5.6 | 8.9 | 6.7 |
| **hsa-miR-181a-5p** | 8106.2 | 9397.7 | 6883.0 | 10624.1 | 14263.3 | 29229.6 | 4910.2 | 5668.5 | 3194.5 |
| **hsa-miR-181b-5p** | 1385.0 | 1273.7 | 1346.5 | 548.6 | 680.1 | 1306.9 | 130.6 | 159.7 | 88.8 |
| **hsa-miR-181c-5p** | 1640.4 | 2288.2 | 2344.6 | 47.4 | 55.8 | 99.8 | 273.5 | 282.4 | 155.6 |
| **hsa-miR-181d** | 488.3 | 605.3 | 933.4 | 6.9 | 9.7 | 14.0 | 21.4 | 18.4 | 9.6 |
| **hsa-miR-18a-5p** | 2601.9 | 2048.6 | 3288.1 | 44.9 | 59.2 | 130.7 | 31.7 | 56.2 | 55.0 |
| **hsa-miR-18b-5p** | 16.8 | 7.9 | 32.7 | n.d. | 0.6 | 0.5 | 0.6 | n.d. | n.d. |
| **hsa-miR-19a-3p** | 2912.3 | 3130.8 | 3222.1 | 271.1 | 172.2 | 363.9 | 309.6 | 548.8 | 355.4 |
| **hsa-miR-19b-3p** | 14014.3 | 13599.7 | 14446.7 | 1955.6 | 1578.5 | 3462.5 | 3834.3 | 6223.5 | 3572.3 |
| **hsa-miR-20a-3p** | 92.5 | 119.4 | 131.4 | 3.6 | 6.9 | 8.2 | 1.9 | 8.2 | 4.7 |
| **hsa-miR-20a-5p** | 24256.9 | 23659.5 | 28264.2 | 480.5 | 700.9 | 973.4 | 474.4 | 870.5 | 650.3 |
| **hsa-miR-20b-5p** | 33.3 | 33.9 | 24.9 | n.d. | n.d. | 1.3 | n.d. | 0.6 | n.d. |
| **hsa-miR-21-3p** | 63317.4 | 116716.1 | 143849.2 | 7729.7 | 12018.4 | 19928.7 | 3870.2 | 5542.4 | 5239.4 |
| **hsa-miR-21-5p** | 641260.9 | 946709.0 | 1255175.0 | 89599.8 | 124414.7 | 93657.1 | 117034.7 | 192900.5 | 184634.5 |
| **hsa-miR-210** | 1635.6 | 1893.1 | 2638.0 | 284.8 | 89.2 | 2467.1 | 1933.0 | 3049.4 | 3884.0 |
| **hsa-miR-211-5p** | n.d. | n.d. | n.d. | 5.9 | 6.9 | 15.5 | 7.8 | 14.1 | 23.2 |
| **hsa-miR-26a-5p** | 390873.7 | 267186.8 | 429762.9 | 111684.4 | 131378.6 | 138644.1 | 155013.3 | 222525.0 | 127611.1 |
| **hsa-miR-26b-5p** | 88027.4 | 57540.1 | 97945.1 | 10837.3 | 13657.4 | 16531.4 | 18200.6 | 25294.5 | 14423.2 |
| **hsa-miR-3177-5p** | 10.1 | 6.2 | 10.3 | n.d. | n.d. | 0.7 | n.d. | n.d. | n.d. |
| **hsa-miR-34a-5p** | 10529.8 | 9204.0 | 11099.2 | 557.7 | 765.9 | 1589.5 | 301.6 | 531.4 | 588.5 |
| **hsa-miR-34b-5p** | n.d. | n.d. | n.d. | n.d. | n.d. | n.d. | 0.7 | 0.6 | 2.3 |
| **hsa-miR-34c-5p** | 1.7 | 4.3 | 1.6 | n.d. | n.d. | n.d. | 33.0 | 47.7 | 138.9 |
| **hsa-miR-3662** | 9.8 | 17.8 | 12.5 | n.d. | n.d. | 6.2 | n.d. | n.d. | n.d. |
| **hsa-miR-3676-3p** | 111.9 | 115.3 | 145.3 | 34.0 | 8.9 | 64.3 | n.d. | n.d. | n.d. |
| **hsa-miR-3676-5p** | 833.9 | 783.5 | 839.6 | 60.4 | 85.3 | 101.4 | n.d. | n.d. | n.d. |
| **hsa-miR-449a-5p** | 2.8 | 1.1 | 1.1 | 1.5 | 2.0 | 3.0 | n.d. | n.d. | 0.7 |
| **hsa-miR-449c-5p** | 7.2 | 4.0 | 2.6 | 0.6 | 0.7 | 3.4 | n.d. | n.d. | n.d. |
| **hsa-miR-455-3p** | 5237.1 | 6795.0 | 7411.8 | 141.3 | 464.0 | 470.1 | 113.8 | 143.4 | 74.1 |
| **hsa-miR-455-5p** | 7270.8 | 7170.9 | 8614.0 | 453.6 | 622.6 | 1057.7 | 74.2 | 101.1 | 55.6 |
| **hsa-miR-500a-3p** | 4848.7 | 5078.6 | 6294.6 | 191.2 | 186.6 | 445.5 | 19.8 | 32.8 | 21.5 |
| **hsa-miR-500b** | 101.6 | 99.7 | 152.5 | 3.9 | 2.6 | 9.5 | n.d. | n.d. | n.d. |
| **hsa-miR-92a-3p** | 821707.4 | 1222186.7 | 1412960.1 | 81588.8 | 129580.5 | 157678.1 | 48270.7 | 65233.2 | 45203.4 |
| **hsa-miR-92b-3p** | 3446.9 | 5840.3 | 10617.3 | 232.8 | 317.2 | 1174.1 | 28.7 | 60.8 | 94.8 |
| **mmu-miR-1199-5p** | n.d. | n.d. | n.d. | n.d. | n.d. | n.d. | 1.7 | 1.4 | 7.7 |
