# Supplementary material for: Integrated analysis of mRNA and miRNA expression in response to interleukin-6 in hepatocytes
Source: Data Brief. 2015 Jun 10;4:226–8. doi: 10.1016/j.dib.2015.05.023 (PMC4510544; doi:10.1016/j.dib.2015.05.023)
Supplement: Supplementary file 1 — Supplementary data [file mmc1.zip › Supplementary Table 2.docx]

**Table 2.** Significant miRNAs binding to over-represented 8nt motif in up- or down-regulated DE mRNA targets

| **Cell type** | **Up-regulated mRNA targets** | **p-value** | **Down-regulated mRNA targets** | **p-value** |
| --- | --- | --- | --- | --- |
| HepG2 | hsa-miR-3177-5p | 2.44E-04 | hsa-miR-181a | 2.20E-02 |
|  | hsa-miR-455-5p | 3.30E-03 | hsa-miR-19a/b | 2.33E-02 |
|  | hsa-miR-19a/b | 1.22E-02 | hsa-miR-455-3p | 4.10E-02 |
|  | hsa-miR-126* | 3.71E-02 | - | - |
|  |  |  |  |  |
| Human  hepatocytes | hsa-miR-455-3p | 2.93E-03 | hsa-miR-3177-5p | 1.22E-03 |
|  | hsa-miR-1286 | 6.10E-03 | hsa-miR-18a/b | 6.61E-03 |
|  | hsa-miR-3177-5p | 1.39E-02 | hsa-miR-92b* | 1.40E-02 |
|  | hsa-miR-20a | 1.79E-02 | hsa-miR-3177-3p | 1.42E-02 |
|  | hsa-miR-17/20b | 1.79E-02 | hsa-miR-455-3p | 1.98E-02 |
|  | hsa-miR-181a | 2.10E-02 | hsa-miR-19a/b | 2.98E-02 |
|  | - | - | hsa-miR-20a | 4.29E-02 |
|  | - | - | hsa-miR-17/20b | 4.29E-02 |
|  |  |  |  |  |
| Mouse  hepatocytes | hsa-miR-455-3p | 2.81E-03 | mmu-miR-126-5p | 2.75E-03 |
|  | mmu-miR-19a/b | 5.86E-03 | hsa-miR-455-3p | 2.93E-03 |
|  | hsa-miR-1286 | 6.10E-03 | hsa-miR-1286 | 5.37E-03 |
|  | hsa-miR-3177-5p | 1.20E-02 | mmu-miR-211 | 8.67E-03 |
|  | hsa-miR-20a | 1.68E-02 | hsa-miR-3177-5p | 1.37E-02 |
|  | hsa-miR-17/20b | 1.68E-02 | hsa-miR-20a | 1.69E-02 |
|  | mmu-miR-455 | 2.05E-02 | hsa-miR-17/20b | 1.69E-02 |
|  | hsa-miR-181a | 2.29E-02 | hsa-miR-181a | 2.32E-02 |
|  | mmu-miR-26a | 3.25E-02 | mmu-miR-500* | 3.68E-02 |
|  | mmu-miR-92b* | 3.64E-02 | mmu-miR-92a/b | 4.15E-02 |
|  | mmu-miR-18a | 4.66E-02 | mmu-miR-19a*/b* | 4.42E-02 |
|  | - | - | mmu-miR-20a* | 4.75E-02 |
