# Supplementary material for: Integrated analysis of mRNA and miRNA expression in response to interleukin-6 in hepatocytes
Source: Data Brief. 2015 Jun 10;4:226–8. doi: 10.1016/j.dib.2015.05.023 (PMC4510544; doi:10.1016/j.dib.2015.05.023)
Supplement: Supplementary file 1 — Supplementary data [file mmc1.zip › Supplementary Table 3.docx]

**Table 3.** Hypergeometric analysis of DE miRNAs and their up- or down-regulated, differentially expressed Targetscan-predicted targets

| **miRNA ID** | **HepG2** | | | | | **Human hepatocytes** | | | | | **Mouse hepatocytes** | | | | |
| --- | --- | --- | --- | --- | --- | --- | --- | --- | --- | --- | --- | --- | --- | --- | --- |
|  | **+/- 6h** | **+/- 24h** | **+ 0-6h** | **+ 0-24h** | **+ 6-24h** | **+/- 6h** | **+/- 24h** | **+ 0-6h** | **+ 0-24h** | **+ 6-24h** | **+/- 6h** | **+/- 24h** | **+ 0-6h** | **+ 0-24h** | **+ 6-24h** |
| **Up-regulated DE mRNA targets** | | | | | | | | | | | | | | | |
| hsa-miR-17-20ab | NA | 7.51E-01 | **1.14E-02** | **6.35E-05** | 9.06E-02 | 4.43E-01 | 8.10E-01 | **4.75E-03** | 8.10E-01 | **1.38E-02** | 4.59E-01 | **6.97E-03** | 2.59E-01 | **4.44E-03** | **1.32E-04** |
| hsa-miR-18ab | NA | 2.65E-01 | NA | 5.26E-02 | 2.78E-01 | **4.44E-02** | 3.08E-01 | 1.74E-01 | 3.08E-01 | **4.21E-02** | NA | **4.70E-02** | 1.81E-01 | **1.15E-05** | **1.34E-06** |
| hsa-miR-19ab | 4.03E-01 | 7.37E-01 | 7.42E-02 | **5.00E-04** | 7.56E-02 | **2.69E-03** | 2.03E-01 | **1.34E-04** | 2.03E-01 | **4.86E-02** | **7.42E-05** | **2.43E-07** | **9.25E-05** | **1.49E-08** | **2.06E-06** |
| hsa-miR-92ab | NA | 2.63E-01 | **2.49E-02** | **1.25E-03** | 5.25E-02 | 2.99E-01 | 7.01E-01 | **8.32E-04** | 7.01E-01 | 6.60E-01 | 6.83E-01 | **1.26E-04** | **3.95E-02** | **5.24E-07** | **2.96E-05** |
| hsa-miR-181a | NA | 3.87E-01 | **1.01E-02** | **2.78E-04** | 2.97E-01 | 1.78E-01 | 4.76E-01 | **1.51E-05** | 4.76E-01 | 4.17E-01 | 1.89E-01 | 6.95E-02 | **1.85E-02** | **7.86E-05** | **1.78E-05** |
| hsa-miR-455 | NA | NA | **3.94E-02** | **2.68E-03** | 1.03E-01 | 5.13E-02 | 5.97E-02 | **4.84E-03** | 5.97E-02 | NA | NA | 4.32E-01 | 2.05E-01 | **3.20E-02** | 5.77E-01 |
| hsa-miR-1286 | NA | NA | NA | 6.47E-01 | 5.76E-01 | NA | NA | 6.95E-01 | NA | NA | NA | 6.73E-01 | NA | 6.16E-02 | 9.32E-02 |
| hsa-miR-3177 | NA | 2.48E-01 | 4.74E-01 | 2.34E-01 | **2.81E-03** | 2.68E-01 | NA | 3.83E-01 | NA | NA | NA | 1.30E-01 | NA | 3.22E-01 | 6.32E-01 |
| mmu-miR-19ab | - | - | - | - | - | - | - | - | - | - | **5.82E-03** | **5.48E-05** | **4.61E-03** | **6.30E-09** | **7.77E-07** |
| mmu-miR-26a | - | - | - | - | - | - | - | - | - | - | 6.16E-01 | 7.56E-02 | 3.51E-01 | **5.29E-05** | **3.79E-05** |
| mmu-miR-126-5p | - | - | - | - | - | - | - | - | - | - | 4.45E-01 | **2.69E-02** | 1.42E-01 | **6.18E-04** | **2.71E-03** |
| mmu-miR-211 | - | - | - | - | - | - | - | - | - | - | NA | 6.22E-02 | 1.22E-01 | **7.30E-03** | **7.71E-03** |
|  |  |  |  |  |  |  |  |  |  |  |  |  |  |  |  |
| **Down-regulated DE mRNA targets** | | | | | | | | | | | | | | | |
| hsa-miR-17-20ab | NA | NA | 7.28E-01 | 9.33E-01 | NA | NA | 6.60E-01 | **7.67E-03** | 6.60E-01 | NA | NA | 5.84E-01 | 7.05E-02 | 7.97E-01 | 8.75E-01 |
| hsa-miR-18ab | NA | 9.46E-02 | **3.32E-02** | 2.53E-01 | NA | NA | NA | **4.37E-02** | NA | NA | NA | 6.77E-01 | 1.98E-01 | 9.43E-01 | 9.73E-01 |
| hsa-miR-19ab | NA | 3.50E-01 | NA | 7.87E-01 | 3.50E-01 | NA | NA | 2.39E-01 | NA | NA | NA | 6.61E-01 | 6.39E-02 | 9.21E-01 | 9.43E-01 |
| hsa-miR-92ab | NA | 2.79E-01 | 6.12E-01 | 9.59E-01 | 2.79E-01 | NA | 5.43E-01 | **4.16E-02** | 5.43E-01 | NA | NA | 6.32E-01 | 5.16E-01 | 8.05E-01 | 8.26E-01 |
| hsa-miR-181a | NA | 3.55E-01 | NA | 9.87E-01 | NA | NA | 8.18E-02 | 5.30E-02 | 8.18E-02 | 6.36E-01 | NA | 3.09E-01 | 6.70E-02 | 9.32E-01 | 9.51E-01 |
| hsa-miR-455 | NA | NA | NA | 2.83E-01 | NA | NA | NA | 2.08E-01 | NA | NA | NA | 4.62E-01 | NA | 8.72E-01 | 9.02E-01 |
| hsa-miR-1286 | NA | NA | NA | 1.90E-01 | NA | NA | NA | 1.35E-01 | NA | NA | NA | 8.54E-01 | NA | 7.23E-01 | 9.49E-01 |
| hsa-miR-3177 | NA | 8.77E-02 | NA | NA | NA | NA | NA | **3.59E-02** | NA | NA | NA | 1.68E-01 | 1.84E-01 | 1.29E-01 | 4.22E-01 |
| mmu-miR-19ab | - | - | - | - | - | - | - | - | - | - | NA | 9.00E-01 | **3.66E-02** | 9.48E-01 | 9.84E-01 |
| mmu-miR-26a | - | - | - | - | - | - | - | - | - | - | NA | 4.44E-01 | NA | 6.92E-01 | 9.26E-01 |
| mmu-miR-126-5p | - | - | - | - | - | - | - | - | - | - | NA | 7.52E-01 | NA | 7.79E-01 | 7.23E-01 |
| mmu-miR-211 | - | - | - | - | - | - | - | - | - | - | NA | 9.75E-01 | NA | 7.20E-01 | 9.22E-01 |
