# Supplementary material for: Integrated analysis of mRNA and miRNA expression in response to interleukin-6 in hepatocytes
Source: Data Brief. 2015 Jun 10;4:226–8. doi: 10.1016/j.dib.2015.05.023 (PMC4510544; doi:10.1016/j.dib.2015.05.023)
Supplement: Supplementary file 1 — Supplementary data [file mmc1.zip › Supplementary Table 4.docx]

**Table 4:** Up-regulated DE mRNA targets of down-regulated DE miRNAs in HepG2 cells

| **hsa-miR-17/20ab** | | **hsa-miR-181a** | | **hsa-miR-455** | | **hsa-miR-19ab** | |
| --- | --- | --- | --- | --- | --- | --- | --- |
| **+ 0-6h** | **+ 0-24h** | **+ 0-6h** | **+ 0-24h** | **+ 0-6h** | **+ 0-24h** | **+ 0-6h** | **+ 0-24h** |
| ADAMTSL2 | APCDD1 | BHLHE40 | ADCY9 | ELF3 | EIF2C4 | BAMBI | ADCY9 |
| BAMBI | BAMBI | FOS | BHLHE40 | GRB10 | ELF3 | FURIN | BAMBI |
| FURIN | BNIP3L | GRB10 | CAPRIN2 | PAQR8 | ETV6 | GRB10 | C8ORF4 |
| RASD1 | C1ORF63 | NRP1 | CCDC92 |  | MOSPD1 | ID2 | DHRS3 |
| SEMA4B | CAPRIN2 | SERPINE1 | CLMN |  | MTUS1 | IL1R1 | DOCK4 |
| SMAD7 | CCNG2 | SLC2A3 | CPT1A |  | PAQR8 | TGM2 | ERBB3 |
| TMCC1 | CSGALNACT1 | SMAD7 | DOCK4 |  | PNPLA6 |  | FURIN |
| UBASH3B | DOCK4 | TMCC1 | EIF2C4 |  | RICTOR |  | GABARAPL1 |
|  | ERBB3 |  | ENPP1 |  | SEMA6A |  | HEG1 |
|  | FURIN |  | ETV6 |  |  |  | HOXD1 |
|  | HEG1 |  | FAM135A |  |  |  | ID2 |
|  | MAP3K8 |  | FOS |  |  |  | IL1R1 |
|  | MARK4 |  | HOXD1 |  |  |  | INSIG2 |
|  | NCEH1 |  | KLF6 |  |  |  | MBNL2 |
|  | NFAT5 |  | MBNL2 |  |  |  | MTUS1 |
|  | PCMTD1 |  | MOSPD1 |  |  |  | PLAG1 |
|  | PLAG1 |  | NFAT5 |  |  |  | PNRC1 |
|  | RASD1 |  | NRP1 |  |  |  | RICTOR |
|  | RBM24 |  | PLAG1 |  |  |  | RORA |
|  | RORA |  | RORA |  |  |  | SYBU |
|  | SLC22A23 |  | SCN9A |  |  |  | TACC1 |
|  | SMAD7 |  | SLC38A2 |  |  |  | TGIF1 |
|  | TACC1 |  | SMAD7 |  |  |  | TGM2 |
|  | TBX3 |  | TMCC1 |  |  |  | TP53INP1 |
|  | TMCC1 |  | WSB2 |  |  |  |  |
|  | TP53INP1 |  |  |  |  |  |  |
|  | YPEL2 |  |  |  |  |  |  |
