# Supplementary material for: Integrated analysis of mRNA and miRNA expression in response to interleukin-6 in hepatocytes
Source: Data Brief. 2015 Jun 10;4:226–8. doi: 10.1016/j.dib.2015.05.023 (PMC4510544; doi:10.1016/j.dib.2015.05.023)
Supplement: Supplementary file 1 — Supplementary data [file mmc1.zip › Supplementary Table 5.docx]

**Table 5:** Up-regulated DE mRNA targets of down-regulated DE miRNAs in human primary hepatocytes.

| **hsa-miR-17/20ab** | | **hsa-miR-181a** | | **hsa-miR-455** | | **hsa-miR-19ab** | |
| --- | --- | --- | --- | --- | --- | --- | --- |
| **+ 0-6h** | **+ 0-24h** | **+ 0-6h** | **+ 0-24h** | **+ 0-6h** | **+ 0-24h** | **+ 0-6h** | **+ 0-24h** |
| ABCA1 | SEMA4B | ADAMTS1 | ETNK1 | CCDC85C | ELF3 | ABCA1 | ETNK1 |
| ACVR1B |  | ADM | ETV6 | CSRNP1 | ETV6 | AKAP7 | RUFY3 |
| DLC1 |  | AKAP7 |  | ELF3 |  | B4GALT5 | SGK1 |
| FAM59A |  | ETNK1 |  | ETV6 |  | BCL3 |  |
| LPGAT1 |  | ETS1 |  | MTUS1 |  | DLC1 |  |
| LRIG1 |  | ETV6 |  | SLC39A14 |  | ETNK1 |  |
| MIDN |  | FAM135A |  |  |  | FAM59A |  |
| OBFC2A |  | KANK1 |  |  |  | IL1R1 |  |
| PFKFB3 |  | LPCAT1 |  |  |  | LPGAT1 |  |
| SEMA4B |  | LPGAT1 |  |  |  | LRIG1 |  |
| STAT3 |  | NRP1 |  |  |  | MTUS1 |  |
| TANC1 |  | PHACTR2 |  |  |  | PFKFB3 |  |
| TNFAIP1 |  | PLDN |  |  |  | PNRC1 |  |
| ZNF295 |  | PRKCE |  |  |  | RUFY3 |  |
|  |  | SIPA1L2 |  |  |  | SGK1 |  |
|  |  | TNFAIP1 |  |  |  | SGMS2 |  |
|  |  | ZFP36L2 |  |  |  | STRN3 |  |
|  |  | ZNF292 |  |  |  |  |  |
|  |  | ZNF295 |  |  |  |  |  |
