# Supplementary material for: Integrated analysis of mRNA and miRNA expression in response to interleukin-6 in hepatocytes
Source: Data Brief. 2015 Jun 10;4:226–8. doi: 10.1016/j.dib.2015.05.023 (PMC4510544; doi:10.1016/j.dib.2015.05.023)
Supplement: Supplementary file 1 — Supplementary data [file mmc1.zip › Supplementary Table 6.docx]

**Table 6:** Up-regulated DE mRNA targets of down-regulated DE miRNAs in mouse primary hepatocytes.

| **hsa-miR-17/20** | | **hsa-miR-181a** | | **hsa-miR-455** | | **hsa-miR-19ab** | |
| --- | --- | --- | --- | --- | --- | --- | --- |
| **+ 0-6h** | **+ 0-24h** | **+ 0-6h** | **+ 0-24h** | **+ 0-6h** | **+ 0-24h** | **+ 0-6h** | **+ 0-24h** |
| Arl4c | Abca1 | Etv6 | Adcy1 | Etv6 | Adcy1 | Arrdc4 | Abca1 |
| Erbb3 | Adam9 | Gcnt2 | Aff1 |  | Calr | Asap2 | Adcy1 |
| Lpgat1 | Aff1 | Itga6 | Atp11a |  | Cldn3 | Bcl3 | Aff1 |
| Raph1 | App | Lpgat1 | Atp1b1 |  | Etv6 | Erbb3 | Arrdc4 |
| Stat3 | Arl4c | Nek7 | Atp2a2 |  | Fbxl20 | Il1r1 | Asap2 |
|  | Atp11a | Nrp1 | Atp8a1 |  | Galnt7 | Itga6 | Atp11a |
|  | Atxn1 | Tbl1xr1 | Atxn1 |  | Magi1 | Lpgat1 | Atxn1 |
|  | Atxn7l1 |  | B4galt1 |  | Mtus1 | Nek7 | Atxn7l1 |
|  | Bmpr2 |  | Bcl9 |  | Pik3r1 | Pak6 | Bcl3 |
|  | Creb5 |  | Bmpr2 |  | Sorbs3 | Raph1 | Bmpr2 |
|  | Crim1 |  | Calr |  | Tnrc6b | Rhob | Card10 |
|  | Dlc1 |  | Cbx7 |  | Zfp36l1 | Sema6b | Cbx7 |
|  | Ephb4 |  | Ccdc6 |  |  |  | Ccdc6 |
|  | Erbb3 |  | Cd2ap |  |  |  | Cgn |
|  | Fndc3b |  | Clmn |  |  |  | Creb5 |
|  | Ggcx |  | Cpne2 |  |  |  | Crebl2 |
|  | Irf1 |  | Crebl2 |  |  |  | Ctgf |
|  | Lasp1 |  | Crim1 |  |  |  | Cxcl12 |
|  | Lima1 |  | Egr1 |  |  |  | Dhrs3 |
|  | Lpgat1 |  | Etv6 |  |  |  | Dlc1 |
|  | Mgea5 |  | Gcnt2 |  |  |  | Erbb3 |
|  | Mkl2 |  | Hsp90b1 |  |  |  | Fam114a1 |
|  | Mllt6 |  | Hyou1 |  |  |  | Fndc3b |
|  | Nav2 |  | Irs2 |  |  |  | Frk |
|  | Nedd4l |  | Itga6 |  |  |  | Igfbp3 |
|  | Pfkp |  | Lpgat1 |  |  |  | Il1r1 |
|  | Pik3r1 |  | Lpp |  |  |  | Inhbb |
|  | Pou6f1 |  | Mfsd6 |  |  |  | Itfg3 |
|  | Rapgef4 |  | Mmp14 |  |  |  | Itga6 |
|  | Raph1 |  | Nek7 |  |  |  | Lpgat1 |
|  | Rhoc |  | Nrp1 |  |  |  | Lphn2 |
|  | Sh3pxd2a |  | Ogt |  |  |  | Lpp |
|  | Slc4a4 |  | Ppap2b |  |  |  | Macf1 |
|  | Sox4 |  | Prox1 |  |  |  | Mfsd6 |
|  | Srgap3 |  | Rnf169 |  |  |  | Mkl2 |
|  | Tacc1 |  | Slc7a11 |  |  |  | Mlec |
|  | Tnrc6b |  | Srgap2 |  |  |  | Mllt6 |
|  | Tspan9 |  | Stk10 |  |  |  | Mtus1 |
|  | Vasp |  | Tbc1d2b |  |  |  | Nek7 |
|  | Ypel2 |  | Tbl1xr1 |  |  |  | Nfia |
|  | Zbtb4 |  | Tcf7l2 |  |  |  | Pak6 |
|  |  |  | Tgfbr1 |  |  |  | Pnrc1 |
|  |  |  | Tmem165 |  |  |  | Rapgef4 |
|  |  |  | Tnrc6b |  |  |  | Raph1 |
|  |  |  | Tns3 |  |  |  | Rhob |
|  |  |  | Zbtb4 |  |  |  | S1pr2 |
|  |  |  | Zfp36l1 |  |  |  | Sdc1 |
|  |  |  |  |  |  |  | Sema6b |
|  |  |  |  |  |  |  | Sh3pxd2a |
|  |  |  |  |  |  |  | Slc31a2 |
|  |  |  |  |  |  |  | Sox4 |
|  |  |  |  |  |  |  | Srgap2 |
|  |  |  |  |  |  |  | Srgap3 |
|  |  |  |  |  |  |  | Stat2 |
|  |  |  |  |  |  |  | Tacc1 |
|  |  |  |  |  |  |  | Tnrc6b |
|  |  |  |  |  |  |  | Zbtb4 |
