# Supplementary figures and images for: Integrated analysis of mRNA and miRNA expression in response to interleukin-6 in hepatocytes
Source: Data Brief. 2015 Jun 10;4:226–8. doi: 10.1016/j.dib.2015.05.023 (PMC4510544; doi:10.1016/j.dib.2015.05.023)

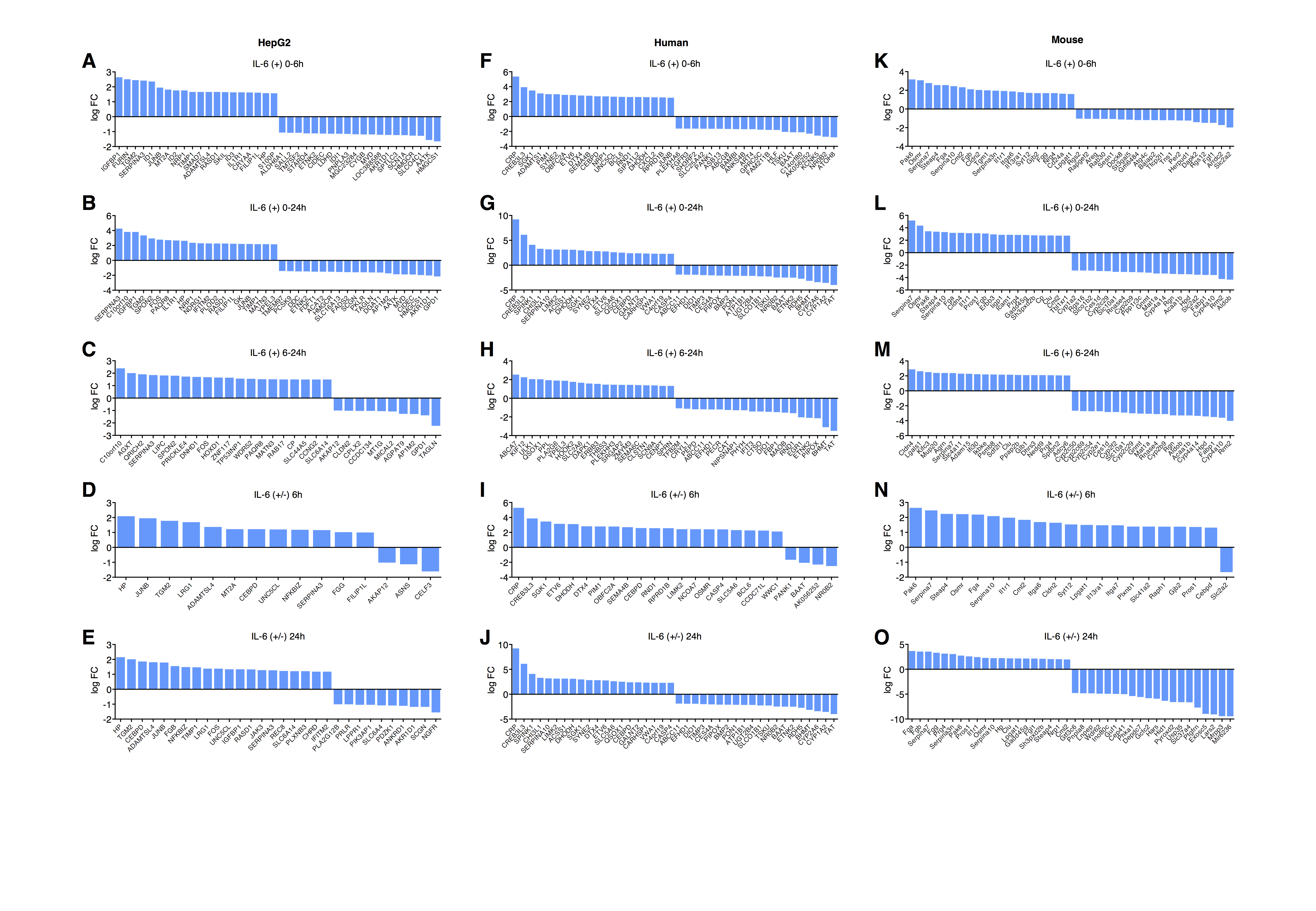

Supplement: Supplementary file 1 — Supplementary data [file mmc1.zip › Supplementary Figure 1.tiff]

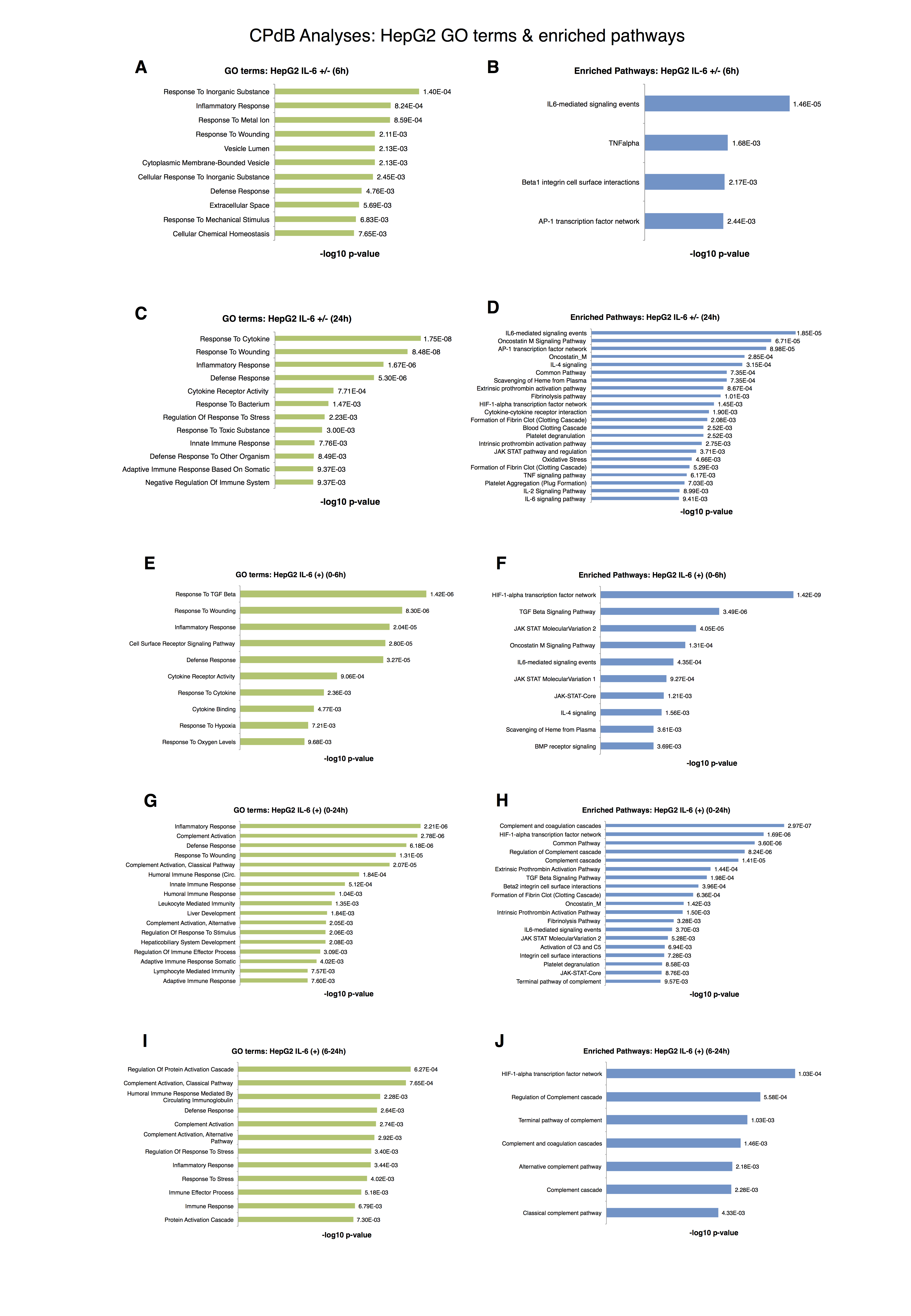

Supplement: Supplementary file 1 — Supplementary data [file mmc1.zip › Supplementary Figure 2.tiff]

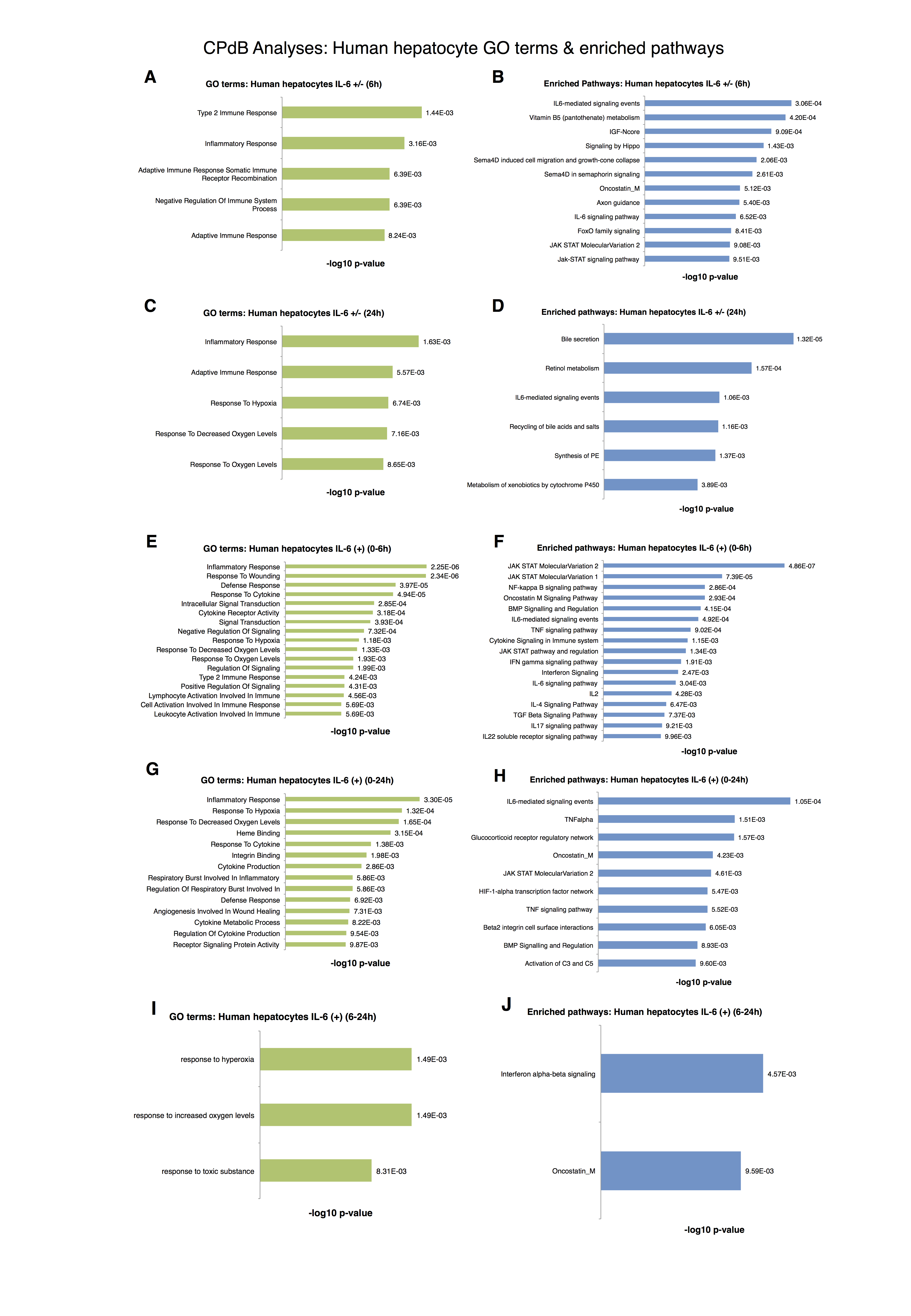

Supplement: Supplementary file 1 — Supplementary data [file mmc1.zip › Supplementary Figure 3.tiff]

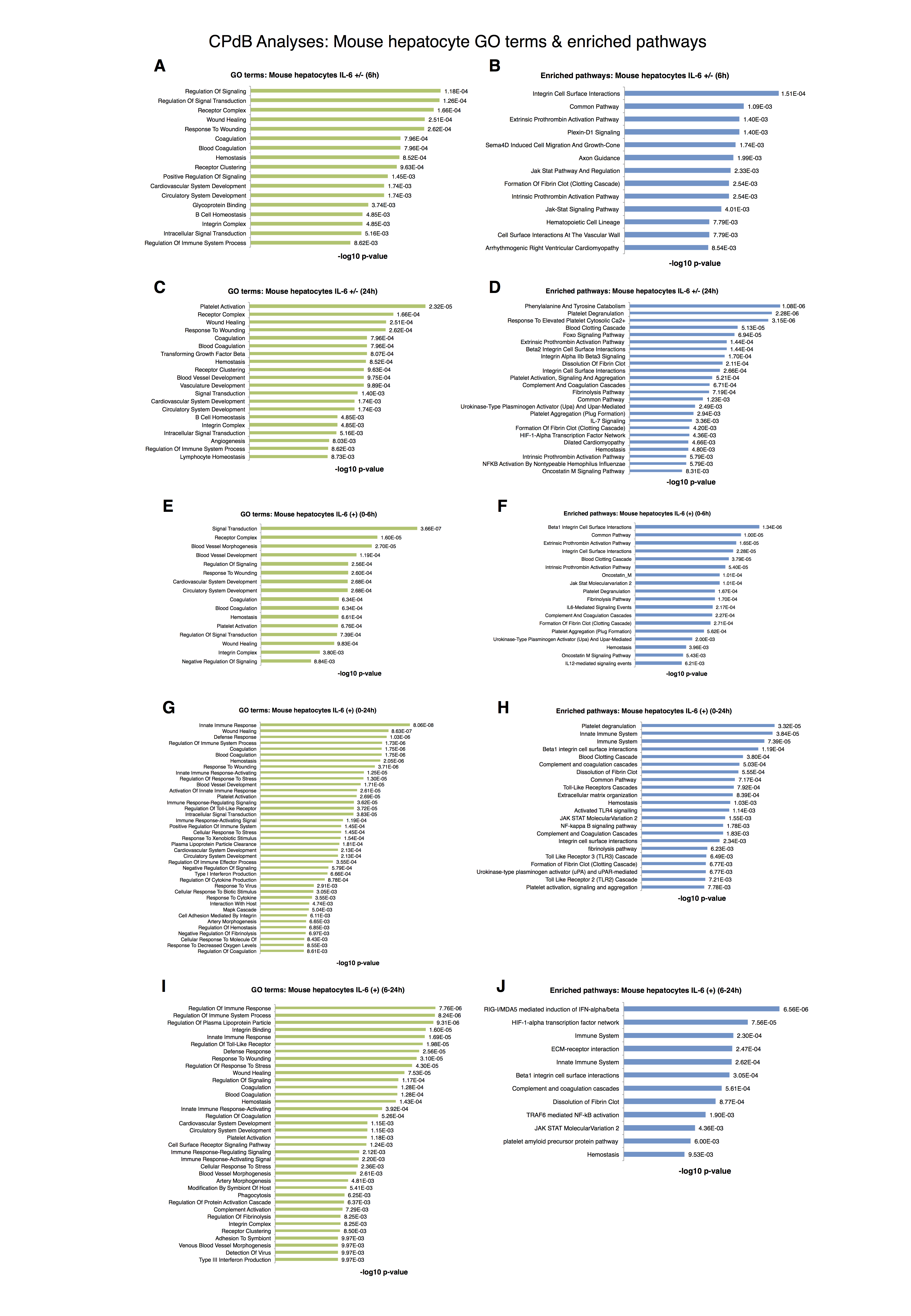

Supplement: Supplementary file 1 — Supplementary data [file mmc1.zip › Supplementary Figure 4.tiff]

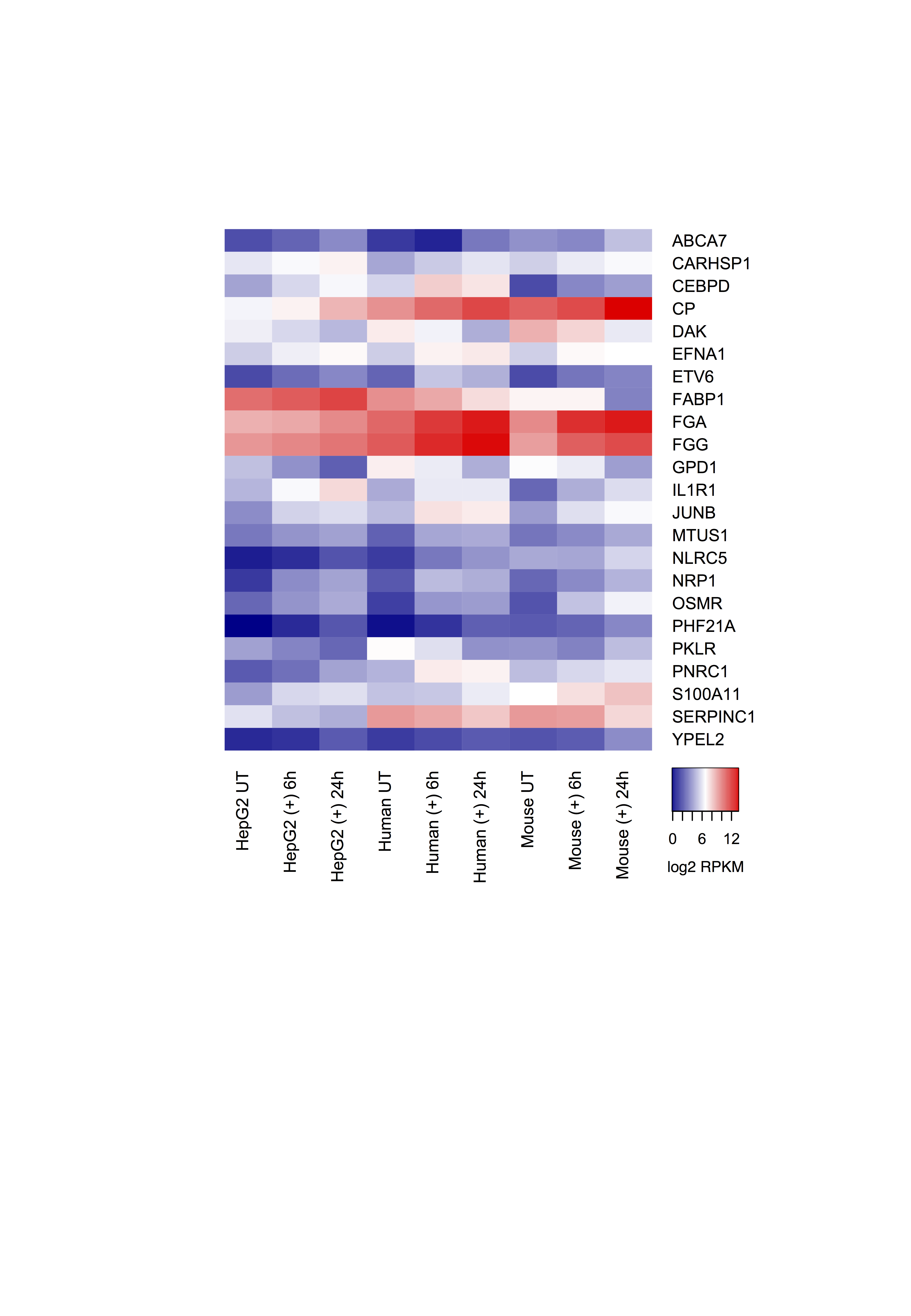

Supplement: Supplementary file 1 — Supplementary data [file mmc1.zip › Supplementary Figure 5.tiff]

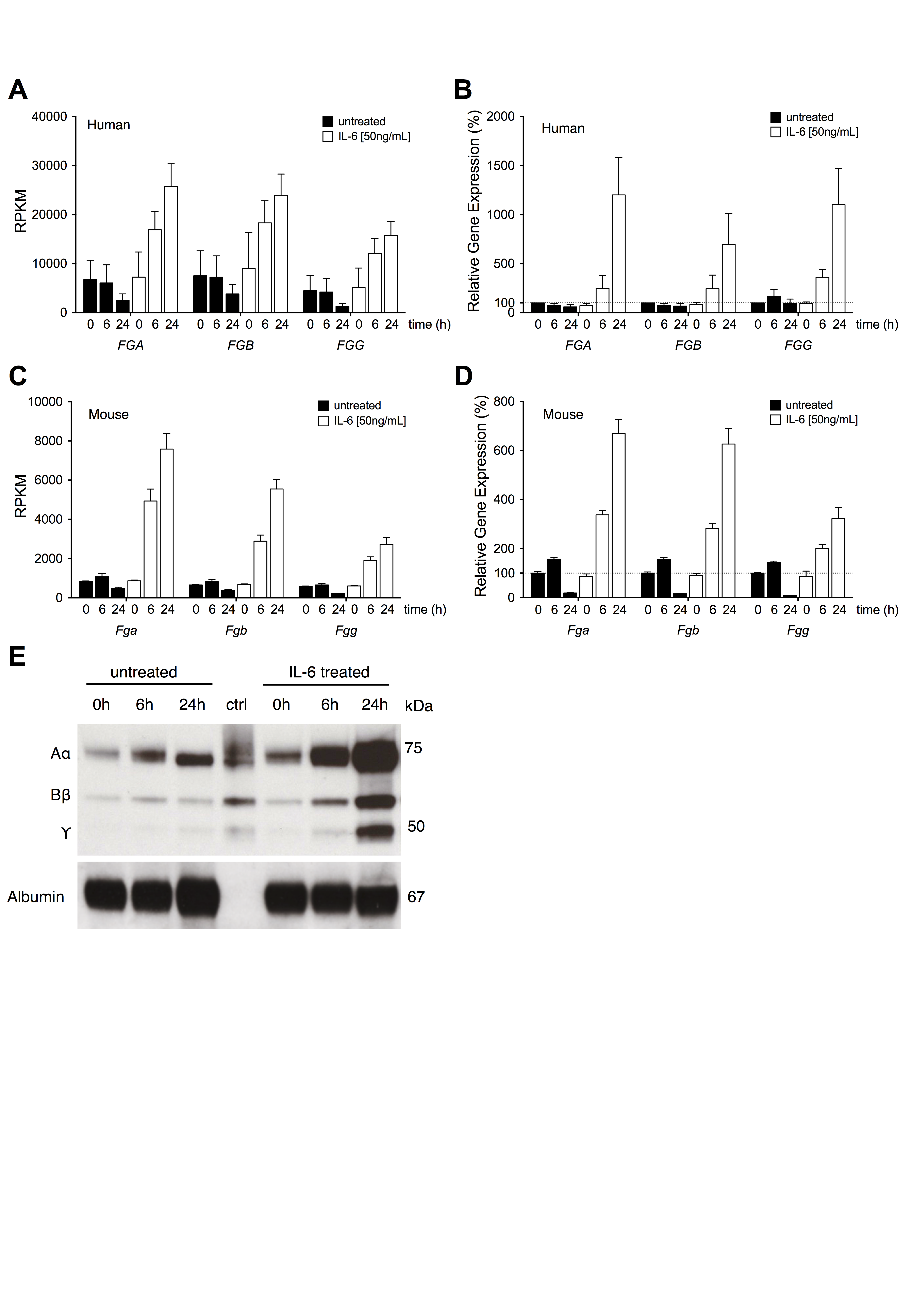

Supplement: Supplementary file 1 — Supplementary data [file mmc1.zip › Supplementary Figure 6.tiff]
